# Study and Realization of Dual-Mode Mobile Light Detection and Ranging Measurement System

**DOI:** 10.3390/s25092679

**Published:** 2025-04-24

**Authors:** Cai Chen, Xiangling Wu, Ming Guo, Xian Ren, Yuquan Zhou, Dengke Li, Liqiong Liao, Zitian Li

**Affiliations:** 1 School of Automation and Electrical Engineering, Zhejiang University of Science and Technology, Hangzhou 310023, China; chencai@zust.edu.cn; 2 Key Institute of Robotics Industry of Zhejiang Province, Hangzhou 310023, China; 3 School of Surveying and Urban Spatial Information, Beijing University of Civil Engineering and Architecture, Beijing 102616, China; 18560519263@163.com (X.W.); 2108160122001@stu.bucea.edu.cn (X.R.); 2108570023111@stu.bucea.edu.cn (Y.Z.); 2108570023125@stu.bucea.edu.cn (D.L.); liaoliqiong@bucea.edu.cn (L.L.); 2108570023120@stu.bucea.edu.cn (Z.L.); 4 Engineering Research Center of Ministry of Education for Representative Building and Ancient Building Data, Beijing 100044, China; 5 Key Laboratory of National Surveying and Mapping Geographic Information Bureau of Modern City Surveying and Mapping, Beijing 100044, China

**Keywords:** mobile LiDAR measurement system, GNSS, navigation and positioning system, time synchronization, point cloud

## Abstract

Most existing mobile LIDAR measurement systems use a GNSS/INS combination method for attitude positioning. This method requires a constant GNSS signal to correct the IMU’s positioning and attitude. In the absence of GNSS signals, the IMU’s positioning accuracy rapidly deteriorates from the centimeter to sub-meter, or even meter levels. To address the positioning limitations of mobile measurement systems without GNSS signals, this paper presents a dual-mode mobile lidar measurement system that combines the GNSS/INS and INS/wheel speed sensor positioning methods, with a time-synchronization controller that automatically switches between the two modes to cope with the loss of GNSS signals. The system uses a high-precision quartz crystal oscillator to simulate the GNSS time data and converts them to an NEMA standard time signal and a PPS signal to synchronize each sensor. The experimental results of the system show that the trajectory error of the dual-mode mobile measurement system reaches 3 m in the X-direction within 600 S compared with that of the ordinary mobile measurement system with GNSS/INS integration mode, and the dual-mode mobile measurement system controls the trajectory error within 1 m, reduces the error in the Y-direction from 2 m to less than 1 m, and reduces the error in the Z-direction from 3 m to less than 2 m. The dual-mode mobile LiDAR measurement system is not only suitable for outdoor road measurement, but also can effectively correct the positioning and attitude errors in the environment without GNSS signals, such as underground and tunnel, showing significant advantages in a variety of measurement scenarios.

## 1. Introduction

The mobile LiDAR measurement system is increasingly becoming a cornerstone of contemporary digital infrastructure development, which consists of measurement sensors, such as the Global Navigation Satellite System (GNSS), the inertial navigation system (INS), and Light Detection And Ranging (LiDAR) [1,2,3]. The deep fusion of the three forms an integrated measurement paradigm of ‘positioning perception’, which provides centimeter-level accuracy spatial information support for urban modelling, traffic survey, and underground space digitization [4,5]. Currently, the mobile lidar measurement system acquires the real-time position and attitude information of the carrier by a combination of GNSS/INS [6,7,8], which relies on the GNSS receiver to observe the satellite data and input them into an Inertial Measurement Unit (IMU), which is used to correct IMU positioning and attitude errors. When the GNSS signal is out of lock, the IMU positioning and attitude accuracy will rise from the centimeter level to the sub-meter level or the even meter level in a very short time [9,10,11]. With respect to the increased measurement error of the combined GNSS/INS navigation approach, even in dual GNSS systems, certain special environments (e.g., tunnels and underground areas) may still result in the loss of GNSS signals. In this case, the dual GNSS system is also unable to provide accurate positioning information. Therefore, a scheme relying solely on the GNSS system cannot comprehensively solve the problem of missing GNSS signals, but instead may cause the system to fail to work stably in certain environments. Although trajectory optimization algorithms [12] and check-and-calibrate methods [13] can improve point cloud accuracy well, in contrast the multimodal sensor fusion architecture improves the robustness and accuracy of the system by integrating data from multiple sensors [14]. Compared to the traditional GNSS/INS combination approach, multimodal systems with additional sensors such as laser radar (LiDAR) can effectively enhance the positioning accuracy [15] and provide strong spatial awareness in complex environments [16]. However, in the absence of GNSS signals, LiDAR may have difficulty in providing sufficient information for precise positioning and attitude estimation. In this paper, a dual-mode autonomous switching mobile LiDAR measurement system is designed for scene measurement with or without a GNSS signal and position fixing with combined GNSS/INS navigation in an area with a good GNSS signal. In the GNSS signal-locked area, a combined INS/wheel speed sensor navigation is used for system positioning and fixing, and the real-time position is calculated by simulating the generation of GNSS time data. The timing synchronization device in this system not only synchronizes time for various sensors [17,18], facilitating the spatial registration of mobile LiDAR point clouds, but also realizes the autonomous switching of two combined navigation modes, which effectively suppresses the dispersion of posture accuracy and improves point cloud accuracy. The proposed dual-mode mobile LiDAR measurement system not only extends the applicability of the mobile LiDAR measurement system in different application scenarios, but also contributes to technological progress in the fields of unmanned driving, automated navigation, and urban infrastructure construction. By solving the positioning problem when the GNSS signal is unstable or lost, the system provides new ideas and directions for future efficient measurements in complex environments.

## 2. Materials and Methods

The design of this dual-mode mobile measurement system mainly considers the design principle of dual-mode time-synchronous mobile measurement systems, the design of a time-synchronous communication module between each sensor, and a design for the automatic switching of dual-mode time-synchronous information to ensure the implementation of the dual-mode mobile LiDAR measurement system.

### 2.1. Design Principle of Dual-Mode Time-Synchronous Mobile Measurement System

**Figure 1:**
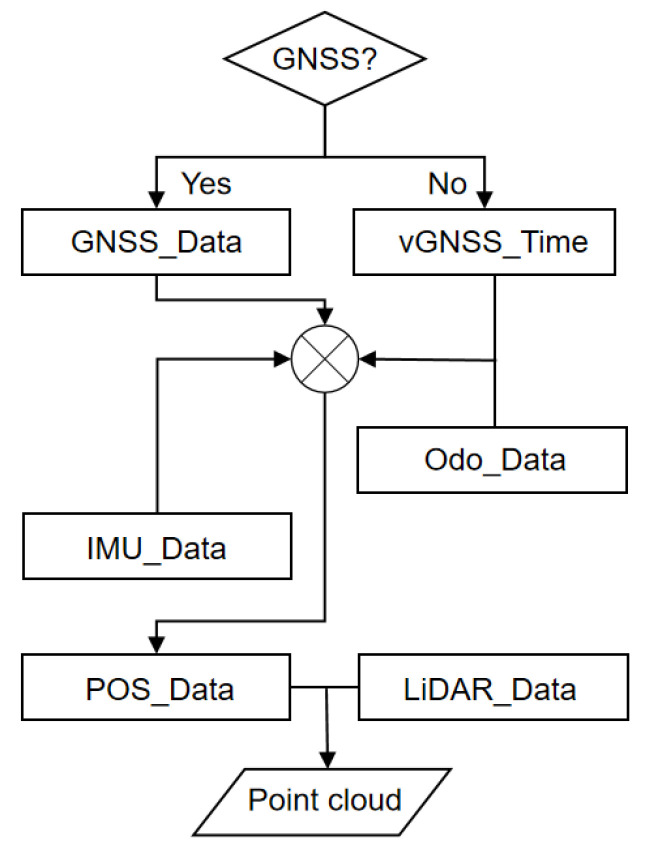
Working principle of dual-mode mobile measurement system.

Dual mode in the dual mode mobile measurement system refers to two different combined navigation modes, and the system can automatically switch between the two combined navigation modes, GNSS/INS and INS/wheel speed sensors, depending on the presence of external GNSS signals. The dual-mode mobile measurement system uses a time-synchronized control module to switch the combined navigation modes, as shown in Figure 1.

The system detects the presence of an external GNSS signal through the time synchronization control module. When the GNSS signal is valid, the system uses the time data provided by the GNSS to synchronize each sensor, thus ensuring that all the sensors work under the same time reference. And it enters combined GNSS/INS navigation mode to output a combination of GNSS data and IMU data as trajectory data. When the GNSS signal is lost, the time synchronization control module uses the built-in high-precision quartz crystal oscillator to continuously provide a high-precision time reference by simulating the GNSS signal. This time reference generates virtual GNSS string time information (vGNSS_Time) and PPS for the time synchronization of each sensor. Even in the absence of GNSS signals, the system relies on IMU data and wheel speed sensor data for trajectory output to ensure that the system is still able to accurately track the state of the motion in the absence of GNSS signals. Combined with the radar data, the final point cloud data are generated to provide rich spatial information for subsequent analysis. Interaction and collaboration between the modules occur through a set of strictly defined interfaces for real-time data exchange and seamless connection. These interfaces ensure that data transfer between the different modules meets the predefined time synchronization and data format requirements, thus realizing the precise collaboration of the system in different operating states.

### 2.2. Dual-Mode Time Synchronization Communication Module Design

**Figure 2:**
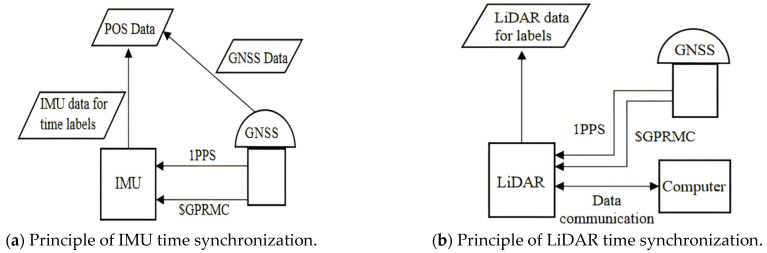
Sensor time synchronization principle.

**Figure 3:**
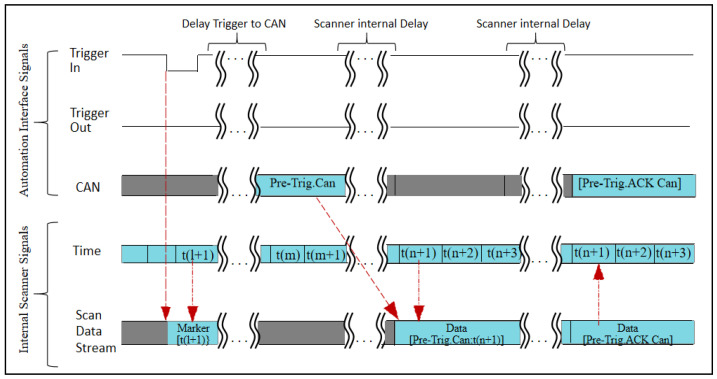
Time synchronization chart.

The working frequency of each sensor of mobile measurement system varies greatly, among which the GNSS working frequency is usually 100 HZ, the INS working frequency is 200 HZ, while the pulse frequency of LiDAR can be as high as 1000 KHZ, so a strict time reference needs to be established to unify the time of each sensor for the splicing of the mobile LiDAR point cloud and trajectory data. A GNSS receiver can obtain accurate GPS time from outside, so the mobile measurement system of GNSS/INS combined positioning and fixing usually uses GPS time as the time reference of the system. The time synchronization principle of the sensor IMU and LiDAR is shown in Figure 2, which generally receives the external GNSS data through the GNSS receiver, including the second pulse PPS signal and the GPS time data, as well as the GNSS receiver position observation value data, and the sensor receives the second PPS and time data from the outside. Then, it triggers the sensor’s internal switches, and the time synchronization circuit inside the sensor activates the internal clock module to ensure that the time reference of the sensor is synchronized with the GNSS clock according to the received PPS signal and the GPS time data as illustrated to obtain the sensor data with time information. A sensor time synchronization timing diagram is shown in Figure 3.

**Figure 4:**
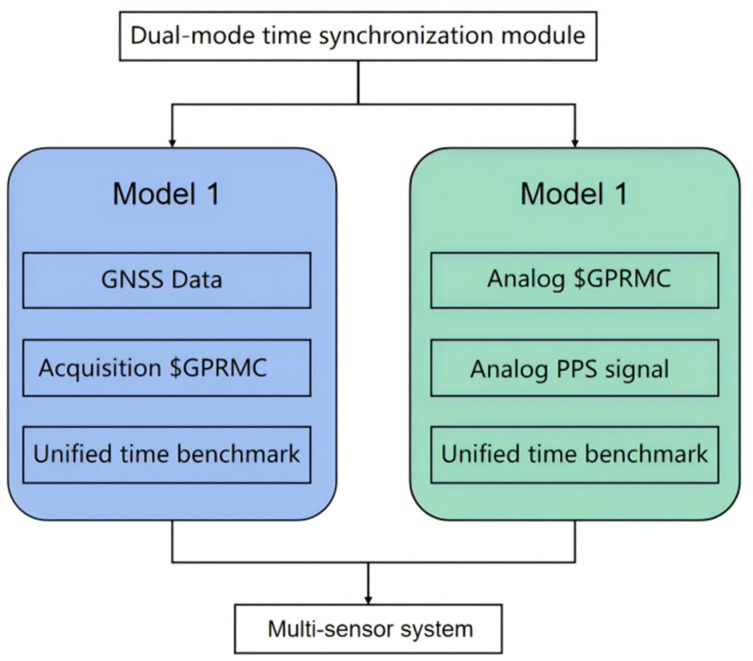
Principle of dual-mode time synchronization.

However, when the conventional mobile measurement system works in a tunnel, under a viaduct, or other areas, it will lose the time reference, causing the failure of multi-sensor time synchronization due to the GNSS signal being out of lock. Then, in the absence of GNSS signal correction in a short period of time, the accumulated error rises rapidly; at this time, the error of the trajectory data recorded in GNSS/INS positioning and navigation mode increases significantly. For this purpose, a dual-mode time synchronization device that can switch the time synchronization mode autonomously is designed. When there is an external GNSS signal, the device synchronizes the time of each sensor by receiving GNSS data from the GNSS receiver; when there is no external GNSS signal, the device utilizes its internal precision clock to autonomously generate simulated $GPRMC time data and PPS data for synchronizing the time of each sensor. As shown in Figure 4, the dual-mode time synchronization device is tested to achieve high-precision time synchronization when no external GNSS signal is available.

### 2.3. Automatic Switching of Dual-Mode Time Synchronization Modules

**Figure 5:**
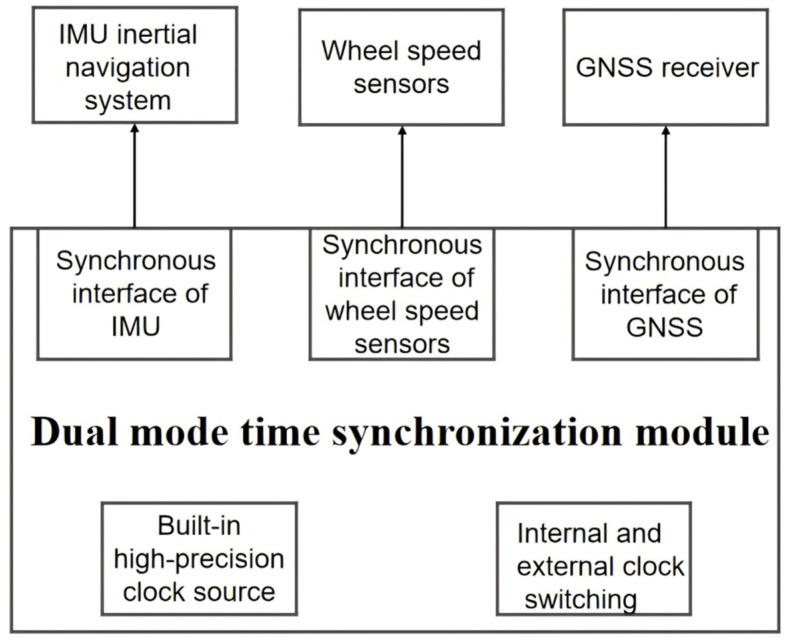
Design of multi-sensor data communication.

The system design of the dual-mode time synchronization module can become compatible with an external GNSS clock (supports GPS/BD) and an internal high-precision standard clock source using a built-in high-performance clock-switching circuit. The number of PPSs determines the quality of the GNSS signal. When the number of PPSs is less than 20 per minute seconds, this is determined as a low-quality GNSS signal, so it automatically switches to the internal clock synchronization mode. When the internal precision clock and GPS time are synchronized, the switching time must be less than 1 us to ensure the accuracy of the data. This principle is shown in Figure 5.

At the same time, the dual-mode time synchronization controller has an inertial navigation system synchronization interface and a wheel speed sensor synchronization interface to achieve IMU and wheel speed sensor data synchronization. The data from the IMU and the wheel speed sensor acquired at the same moment are jointly computed to obtain the IMU position and attitude data. According to the determination of GNSS signal quality, the dual-mode time synchronization control module achieves the rapid, automatic switching of the two positioning navigation modes to ensure that each sensor’s signal and data are transmitted effectively.

## 3. Experiments

The hardware part of the dual-mode mobile measurement system is mainly composed of three modules: a data acquisition module, a positioning and attitude module, a and time synchronization control module. The data acquisition module is an LiDAR sensor; the positioning and attitude module consists of a GNSS receiver (Parameters are shown in Table 1), an IMU, and a wheel speed sensor; and the time synchronization control module is controlled by an embedded microprocessor as the core. The data acquisition module is responsible for 3D point cloud data acquisition; the positioning and attitude module provides continuous posture information for the system; and the time synchronization control module receives and parses the original data from the GNSS receiver, the IMU, and the wheel speed sensor and sends them to the computer and also switches between the different navigation and positioning modes according to the quality of the external GNSS signals. When the GNSS signal is normal, the time synchronization control module realizes the acquisition and forwarding of the GNSS data; when the GNSS signal is out of lock, the time synchronization control module temporarily takes over the GNSS receiver to realize high-precision time synchronization, and the synchronization data fully conform to the NEMA standard, which can completely realize the seamless switching of navigation data.

### 3.1. Time Synchronization Control Module

**Figure 6:**
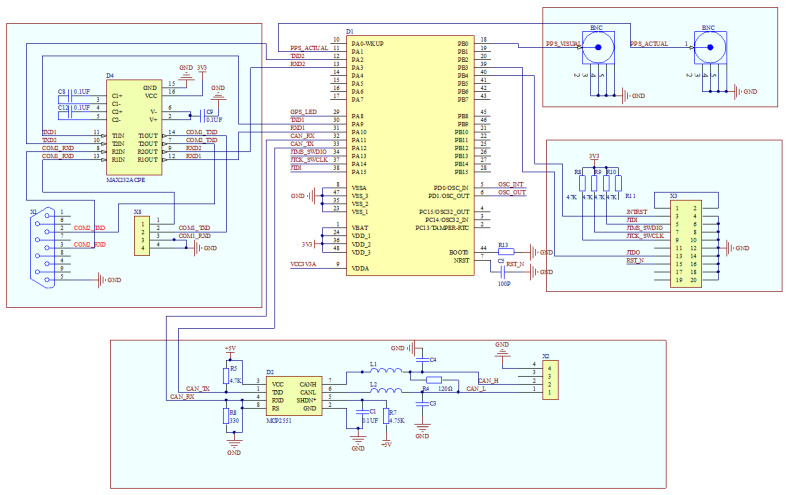
Time synchronization control module schematic.

Microprocessors are required for synchronization mode switching and data transmission in dual-mode time-synchronous mobile measurement systems. The system design uses an ARM high-performance microprocessor, which has a bit width of 32 bits, a frequency of 72 MHz, a flash capacity of 256 KB, and an RAM capacity of 48 K. It has on-chip peripheral resources, including 2 basic timers, 4 general-purpose timers, 2 advanced timers, 5 serial ports, and 51 general-purpose IO ports. A schematic diagram of the time synchronization control module is shown in Figure 6. The control module was developed using an ARM microprocessor, SHDN* indicates that the pin is active low.

**Figure 7:**
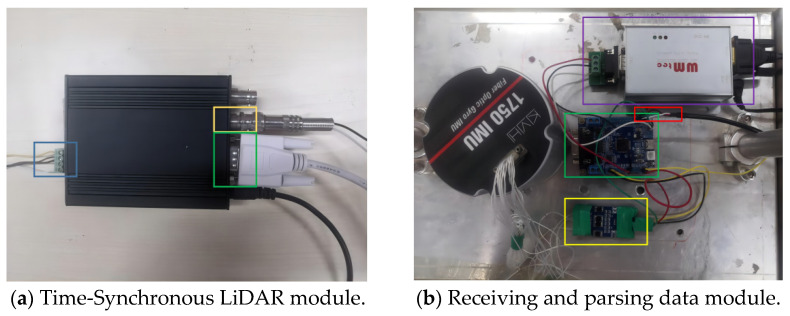
Time synchronization control module.

The time synchronization control module is the time synchronization device and data transmission center of the system. During the operation of the dual-mode mobile measurement system, the microprocessor receives raw data from the IMU, the GNSS receiver, and the wheel speed sensor through a serial port, and the firmware program in the microprocessor parses these data. If the GNSS data can be parsed, it uses GPS time as the time standard for the time synchronization of each sensor and outputs the comprehensive navigation results of GNSS/INS, If the GNSS data are not detected, it will automatically switch to analog GNSS synchronization mode using the high-precision crystal oscillator inside the time synchronization device as the clock source. It also uses this clock as the time standard for time synchronization of each sensor and outputs the combined INS/wheel speed sensor navigation data. Figure 7a shows a physical diagram of the time synchronization communication module, where the $GPRMC time data are input from the RS232 serial port (shown in green box in the figure), the PPS data are input from the serial port (shown in yellow box in the figure), and the data are output from the interface CAN to LiDAR for time synchronization (shown in blue box in the figure). Figure 7b shows a physical wiring diagram of the data receiving and parsing module, in which the GPS time and position data received by the GNSS receiver are connected to the microprocessor (shown in the green box in the figure) by RS232 to TTL (shown in the purple box in the figure), and at the same time, the second pulse PPS data sent by the GNSS receiver trigger the IMU to generate the corresponding bit pose data through the 422-TTL interface (shown in the yellow box in the figure). The data are parsed by the microprocessor firmware program and sent to the computer by the serial port uniformly (shown in the red box in the figure).

### 3.2. Design of Time Synchronization Control Module Firmware Program

**Figure 8:**
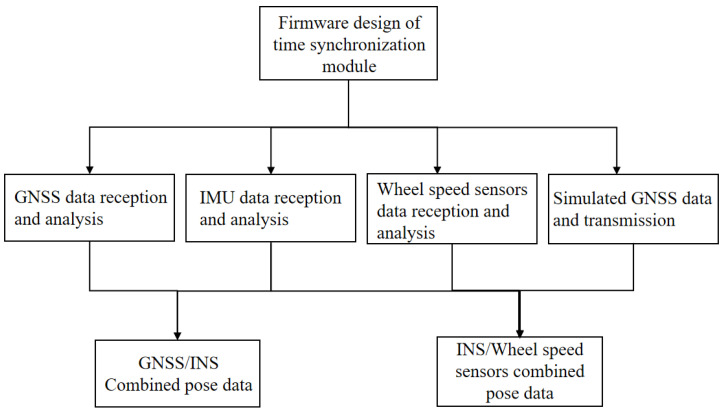
Firmware architecture of dual-mode mobile measurement system.

The program running in the time synchronization control module is a firmware program. The firmware program mainly realizes 2 functions; 1 is to simulate the generation of GNSS time data, and 2 is to realize the reception and parsing of the raw data from each sensor. And the parsed data are combined into GNSS/INS posture data and INS/wheel speed sensor posture data, respectively, according to the synchronization mode of the current system operation. With the microprocessor as the data processing center of the whole system, the software development of the firmware program is carried out using C language, employing embedded development techniques to implement the corresponding firmware functions. The overall firmware program architecture is shown in Figure 8.

#### 3.2.1. Multi-Measurement Sensor Data Reception and Analysis

**Figure 9:**
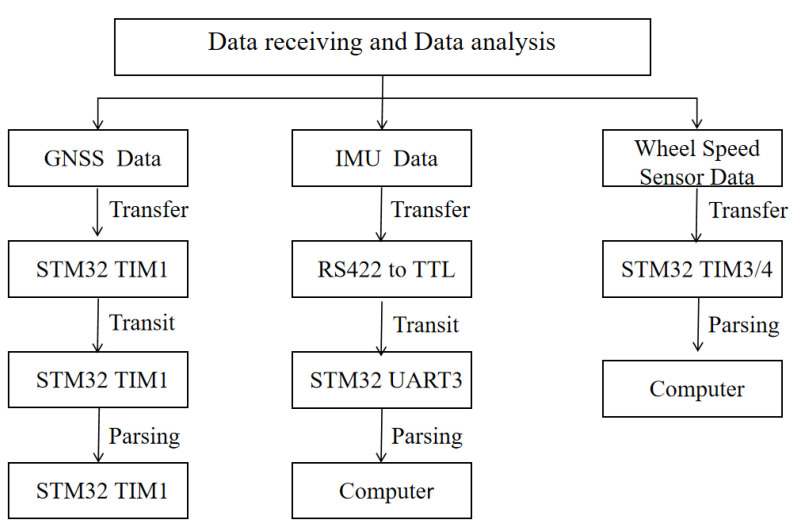
Multi-sensor data receiving and data analysis.

The GNSS data are the basis for the time synchronization of each sensor of the mobile measurement system and are transmitted as per the NMEA 0183 protocol. The statement used in this system is the $GPRMC format statement. The GNSS receiver receives the $GPRMC data, which are then sent to the computer via the time synchronization control module; the baud rate is set to 115,200 bps, and it writes a code to realize the reception and parsing of the GNSS data according to the NEMA protocol. The IMU used in this system adopts an advanced fiber optic gyroscope with the advantages of high bandwidth, low latency, and low drift, and integrates a low-level noise micro-motor accelerometer for the more accurate calculation of line acceleration. This is the smallest high-performance fiber optic gyro sensor in the world, which can still provide accurate position attitude information in an extreme environment and has excellent shock and vibration resistance. It is a six-axis (three-axis linear velocity and three-axis angular velocity) high-precision sensor and sends posture information at the RS422 differential level. The IMU data are 36 bytes, the first 4 bytes are frame headers, fixed at 0xFE81FF55, which will not appear at any position in any frame; the middle 28 bytes are data bits, including angular velocity and linear acceleration; and the last 4 bytes are check bits. The IMU data are received by the serial port of the time synchronization controller through the RS422-TTL module, the baud rate is set to 115,200 bps, and the reception and parsing of IMU data are implemented by the firmware. The wheel speed sensor used in this system supports data transmission via a TTL-level signal; when the system is integrated, the lead is used to directly connect the pulse data output port of the photoelectric encoder to the TIM3 and TIM4 ports of the time synchronization controller, and the code is written according to the working principle of the photoelectric encoder to realize the reception and analysis of the wheel speed sensor data. The data reception and analysis of each sensor are shown in Figure 9.

#### 3.2.2. Simulation Time and PPS Generation

**Figure 10:**
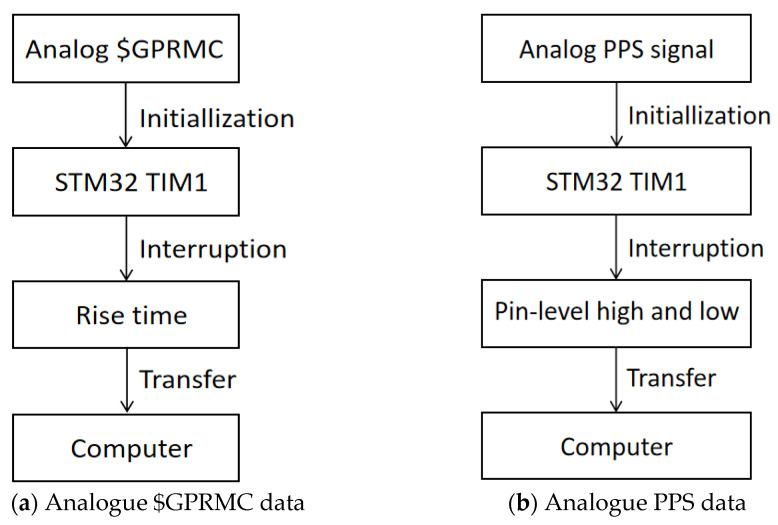
Simulated GNSS data.

A dual-mode mobile measurement system that works properly without GNSS signals requires a time reference for each sensor to synchronize the time of each sensor. The system uses a high-precision timer inside the time synchronization control module to interrupt at a crystal frequency of 72 MHz. The timer pre-distribution coefficient is configured to 9 divisions, with a timing accuracy of 0.1 us or more, so that the time synchronization control module can rely on itself to provide virtual $GPRMC time and PPS data and send a combination of the $GPRMC data and the PPS data to the sensors to be synchronized, which can achieve the purpose of multi-sensor time synchronization. The specific steps of simulating the generation of the $GPRMC and PPS data are shown in Figure 10.

### 3.3. Dual-Mode Mobile LiDAR Measurement System Integration Effect

**Figure 11:**
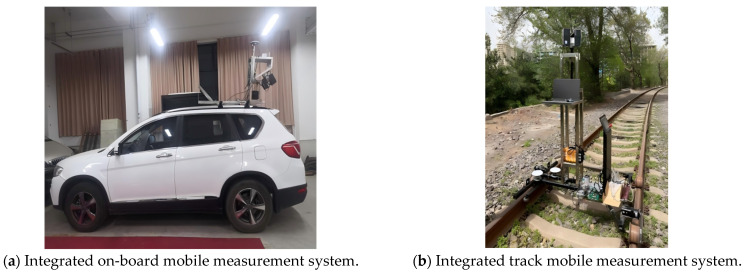
Dual-mode mobile LiDAR system integration.

The time synchronization control module transmits the time signal and the data for the LIDAR module and the attitude positioning module, so that the dual-mode mobile LIDAR measurement system can obtain the track data and the point cloud data at the same moment, and because the dual-mode mobile measurement system adopts two modes of positioning, GNSS/INS positioning and INS/wheel speed sensor positioning, it can automatically switch the positioning mode according to the presence or absence of a GNSS signal input. Therefore, it can adapt to a variety of scenes, measurements, and integrate vehicle measurement systems and track measurement systems according to the different carrying platforms, as shown in Figure 11.

With an ordinary mobile LiDAR measurement system using GNSS/INS combined navigation mode to obtain trajectory data, trajectory errors are addressed based on algorithms when the GNSS signal is out of lock, which leads to its use only over a short time. Once the GNSS signal has been out of lock for a long time, the trajectory may differ. The dual-mode mobile measurement system features two distinct combined navigation methods. By automatically switching between these navigation modes, it utilizes different sensors to acquire the trajectory data, offering a fundamental solution for enhancing the precision of the trajectory data, and is suitable for the case of the GNSS signal being out of lock for a long time. This navigation mode is especially suitable for roads with road bridges, tunnels, and as a part of tunnel point cloud measurement.

## 4. Results

### 4.1. Vehicle-Based Measurement System Trajectory Data Pre-Processing

**Figure 12:**
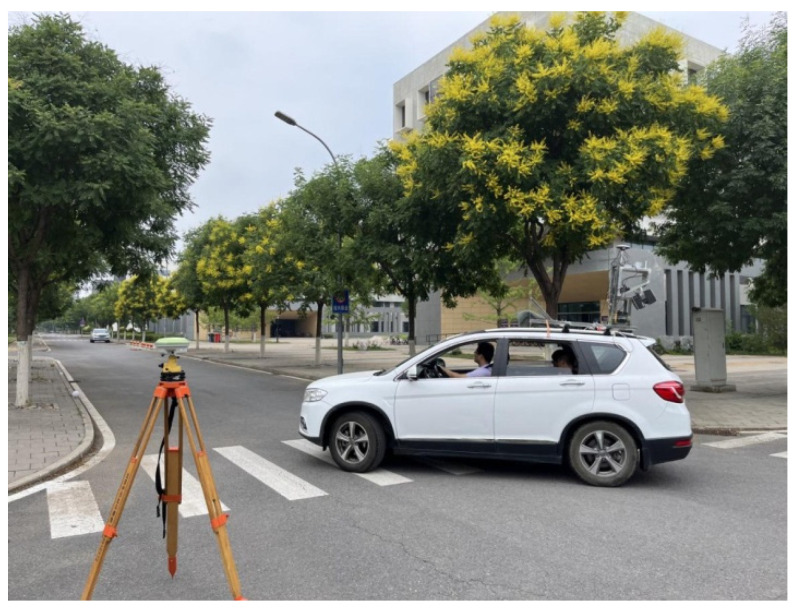
Mobile measurement road test.

The vehicle-based measurement system takes the campus road of Beijing University of Architecture as the experimental site, as shown in Figure 12. The campus is full of trees on both sides of the road. The GNSS signal will appear intermittent in this situation, which is conducive to test the GNSS/INS and INS/wheel speed two-sensor combined navigation and positioning mode automatic switching effect.

**Figure 13:**
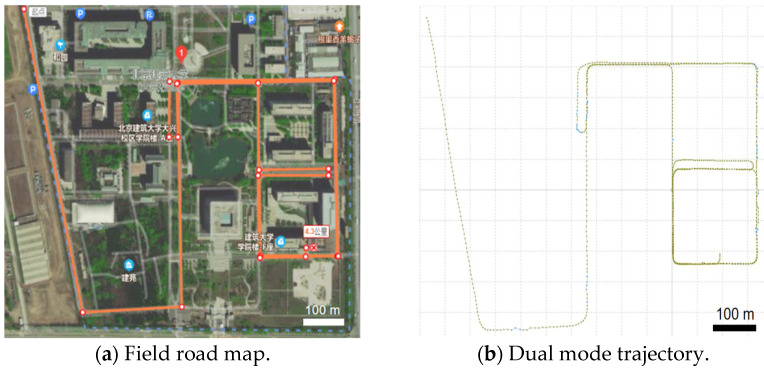
Vehicle-mounted dual-mode mobile LiDAR system measures trajectory.

The vehicle-mounted mobile LiDAR measurement system collects the posture data on the road for each frame, including the GPS time, GNSS receiver position observations, the IMU data, and the wheel speed sensor data. Multiple measurements of the same feature are beneficial to determine whether the trajectory data are accurate. Through experimentally testing the dual-mode mobile LIDAR measurement system, we can stably receive and resolve each sensor data, and the data from the reference station and the mobile station are jointly solved to finally form the trajectory under the Beijing local coordinate system, as shown in Figure 13.

As can be seen from this figure, the trajectory of the vehicle-mounted dual-mode measurement system is consistent with the field road map, and the trajectory positioning is good.

### 4.2. Overall System Accuracy Test

**Figure 14:**
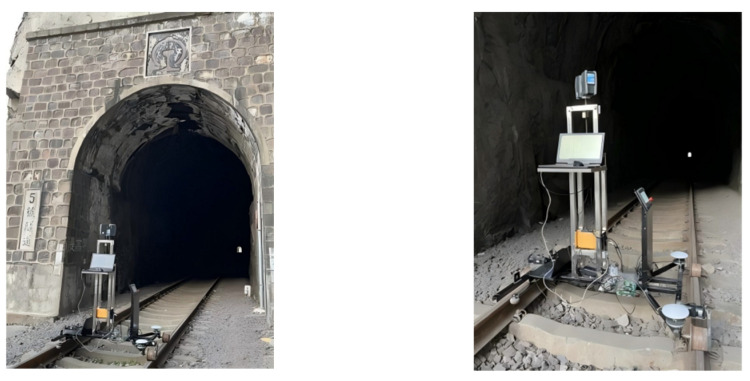
Tunnel test of Jingmen railway.

The experiments were carried out using a dual-mode mobile measurement system with a railcar as the piggyback platform for posture error analysis, and the quality and state of the GNSS signals were tested, respectively. The system was tested as a whole on Jingmen railway, which is 53 km long and has several tunnels midway, meeting the working environment requirements of the track-type dual-mode mobile measurement system. It is convenient to compare and analyze the positioning and fixing accuracy of the two combined GNSS/INS and INS/wheel speed sensor navigation modes. The experimental site is shown in Figure 14.

We conducted three separate overall system tests in the same area. The first group normally connected the GNSS antenna and the hardware to simulate the normal operation of the dual-mode measurement system, which was recorded as Exp1. The second group did not install the GNSS antenna and did not connect the wheel speed sensor, simulating the attitude data collected by the mobile measurement system when working in an environment without a GNSS signal, which was recorded as Exp2. The third group did not install the GNSS antenna, normally connected the other hardware, and simulated the attitude data collected by the dual-mode mobile measurement system when working in an environment without a GNSS signal, which was recorded as Exp3.

### 4.3. Effect of Dual-Mode Mobile Measurement System on Posture Error

**Figure 15:**
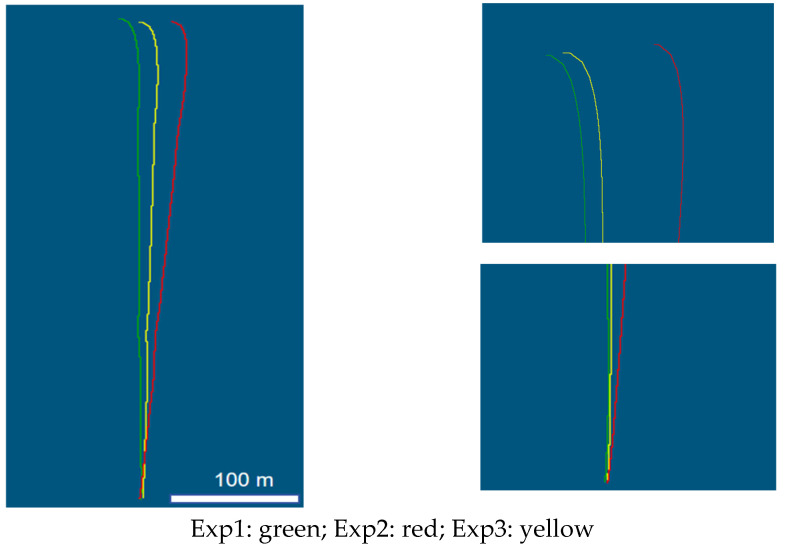
Trajectory data of different experimental groups.

To investigate the influence of the dual-mode mobile measurement system on positional accuracy, the trajectory data obtained in Exp1 are used as the true value, and the trajectory data obtained in Exp2 and Exp3 are used as the reference value for accuracy analysis, and the trajectory data obtained in the three groups of experiments are shown in Figure 15.

**Figure 16:**
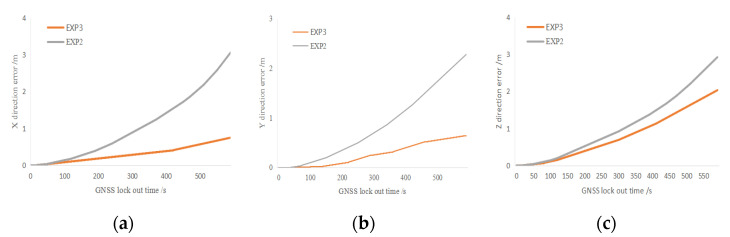
With or without INS/wheel speed sensor error comparison. (**a**) Error distribution of Exp2 and Exp3 on *X* axis. (**b**) Error distribution of Exp2 and Exp3 on *Y* axis. (**c**) Error distribution of Exp2 and Exp3 on *Z* axis.

In Figure 15, the green line is the trajectory data obtained in Exp1, the red line is the trajectory data obtained in Exp2, and the yellow line is the trajectory data obtained in Exp3. It can be seen from the figure that in Exp2, with the increase in system running time, the trajectory data drift phenomenon is obvious, and the error is large. Exp3 demonstrated an obvious suppression effect on the dispersion of positional accuracy, and the trajectory data are closer to the true value. To quantify and analyze the trajectory data of Exp2 and Exp3, we calculated the errors of trajectory in the X-, Y-, and Z-directions and determined the improvement effect of the dual mode motion measurement system on pose accuracy in the absence of the GNSS. The errors of Exp2 and Exp3 in each direction are shown in Figure 16, the quantitative table of error comparison is shown in Table 2.

As can be seen in Figure 16, the dual-mode mobile measurement system has a certain enhancement effect in all the directions. The error of the ordinary GNSS/INS integrated mode mobile measurement system in the X-direction reaches 3 m within 600 s, and the dual-mode mobile measurement system controls the trajectory error within 1 m, reduces the error from 2 m to within 1 m in the Y-direction, and reduces the error from 3 m to within 2 m in the Z-direction. The experiment verifies that the dual-mode mobile LiDAR measurement system effectively improves the trajectory accuracy compared with that of the common mobile measurement system, and thus can improve point cloud accuracy.

## 5. Conclusions

In this study, a dual-mode autonomous switching mobile LiDAR measurement system is developed to address the measurement challenges in environments with unstable GNSS signals. The system uses combined GNSS/INS navigation for positioning and attitude fixing in areas with good GNSS signals. When entering a region with out-of-lock GNSS signals, the system switches to the combined navigation technique of INS and wheel speed sensors, while simulating the generation of GNSS time data for the accurate computation of real-time position. A car and a railcar are used as mobile platforms, respectively, and different scenarios are tested to pre-process the trajectory data, and control experiments are carried out in the track area to test the influence of the mobile measurement system on the trajectory data when accessing the different sensors. The experiments prove that the dual-mode mobile measurement system has a significant effect in improving trajectory accuracy. The key innovation of the system is the design of the time synchronization device, which ensures precise time synchronization between the sensors. Time synchronization is crucial for the spatial alignment of mobile LiDAR point clouds, as time deviations can directly affect the accuracy and reliability of point cloud data. The contribution of this study is to provide a new solution to the position error problem in combined GNSS/INS navigation systems, which is important for improving navigation and position services in complex environments. To further enhance the benefits of the dual-mode system in areas with poor GNSS signals, future research will consider improving accuracy by integrating additional sensors or data sources and exploring more efficient sensor fusion algorithms.

## Figures and Tables

**Table 1:** Technical parameters of GNSS board.

Technical Indicators	Technical Parameters
Antenna type	OEM7720 dual antenna
Position accuracy	Single point L1: 1.5 m
	Single point L1/L2: 1.2 m
	SBAS: 0.6 m
	DGPS: 0.4 m
	RTK: 0.01 m + 1 ppm
Frequency of data updates	Maximum 100 HZ
Time accuracy	20 ns RMS
Velocimetry accuracy	<0.03 m/s
Speed limit	515 m/s
Temperature	−40–85 °C
Humidity	Non-condensing
Communication interface	3 LVCMOS
	2 CAN
	1 USB

**Table 2:** Sensor error comparison quantitative table.

	X (m)	Y (m)	Z (m)
Exp2	3	2.5	3
Exp3	0.8	0.5	2

## Data Availability

The data are available from the author, Xiangling Wu, upon reasonable request.
